# GABA as a rising gliotransmitter

**DOI:** 10.3389/fncir.2014.00141

**Published:** 2014-12-17

**Authors:** Bo-Eun Yoon, C. Justin Lee

**Affiliations:** ^1^Department of Nanobiomedical Science, Dankook UniversityChungnam, South Korea; ^2^WCI Center for Functional Connectomics, Korea Institute of Science and Technology (KIST)Seoul, South Korea; ^3^Center for Neural Science and Center for Functional Connectomics, Korea Institute of Science and Technology (KIST)Seoul, South Korea

**Keywords:** astrocyte, gliotransmitter, glial GABA, tonic inhibition, MAOB

## Abstract

Gamma-amino butyric acid (GABA) is the major inhibitory neurotransmitter that is known to be synthesized and released from GABAergic neurons in the brain. However, recent studies have shown that not only neurons but also astrocytes contain a considerable amount of GABA that can be released and activate GABA receptors in neighboring neurons. These exciting new findings for glial GABA raise further interesting questions about the source of GABA, its mechanism of release and regulation and the functional role of glial GABA. In this review, we highlight recent studies that identify the presence and release of GABA in glial cells, we show several proposed potential pathways for accumulation and modulation of glial intracellular and extracellular GABA content, and finally we discuss functional roles for glial GABA in the brain.

## Introduction

Recent experimental evidence suggests that glial cells interact closely with neurons and play an active role in the brain. In the central nervous system (CNS), astrocytes, the major type of glia, make direct contacts with neurons via a structure that has been defined as the tripartite synapse, in which the astrocytic process is associated with the pre- and post-synapse areas of neurons (Araque et al., 1999). Indeed, astrocytes sense neuronal activity by expressing various receptors for neurotransmitters (Marcaggi and Attwell, 2004; Schipke and Kettenmann, 2004) and they are also able to release various transmitters that modulate neuronal excitability and synaptic transmission (Volterra and Meldolesi, 2005; Perea and Araque, 2010; Zorec et al., 2012). Of the various transmitters released by glial cells, so-called gliotransmitters, most of the excitatory transmitters, glutamate, ATP and D-serine have received much attention (Volterra and Meldolesi, 2005; Fiacco and McCarthy, 2006; Haydon and Carmignoto, 2006; Oliet and Mothet, 2006). However, classical inhibitory transmitters are also released by glial cells and can modulate neuronal activity. Some studies have suggested that taurine and glycine act as gliotransmitters (Barakat and Bordey, 2002; Hussy, 2002) and some initial reports showed an accumulation of GABA by glial cells (Barres et al., 1990; Gallo et al., 1991; Ochi et al., 1993). Despite these early ideas and recent evidence, the principal CNS inhibitory transmitter, GABA had not been considered as a gliotransmitter (Angulo et al., 2008). However, recent studies in primary cultures (Liu et al., 2000), human astrocytes (Lee et al., 2011b) and acute brain slices of various brain regions including thalamus, olfactory bulb and cerebellum (Barakat and Bordey, 2002; Kozlov et al., 2006; Lee et al., 2010; Jiménez-González et al., 2011) have demonstrated a robust release of GABA from glial cells. Moreover, GABA released by glial cells activates the high affinity GABA receptors to mediate tonic inhibition (Lee et al., 2010; Héja et al., 2012; Wójtowicz et al., 2013). Glial cells have thus begun to make their mark not only as GABAceptive cells, but also as GABAergic cells (Yoon et al., 2012). These exciting new findings raise questions about the gliotransmitter release mechanism by glia and the origin and regulation of glial GABA. Furthermore, alteration of tonic inhibition is found in various pathological states such as absence seizures, after strokes and in Huntington’s disease (Clarkson et al., 2010; Pirttimaki et al., 2013; Wójtowicz et al., 2013). Perhaps these changes in tonic inhibition occur through glial GABA, a possibility that awaits further investigation. It has recently been reported that tonic GABA release from reactive astrocytes is enhanced and memory is impaired in mouse models of Alzheimer’s disease (Jo et al., 2014; Wu et al., 2014). From initial reports showing GABA accumulation by glial cells to the more recent studies that give electrophysiological and pathophysiological evidence of a tonic GABA release that is mediated by glial GABA, one can safely conclude that glial GABA modulates neuronal activity and has an important physiological function. In this review, we present an overview of the cellular localization of GABA, its production and release mechanisms, and the potential roles for glial GABA, that all point towards the new concept of GABA as “a rising gliotransmitter” in the brain.

## Cellular localization of glial GABA in various brain regions

**Figure 1:**
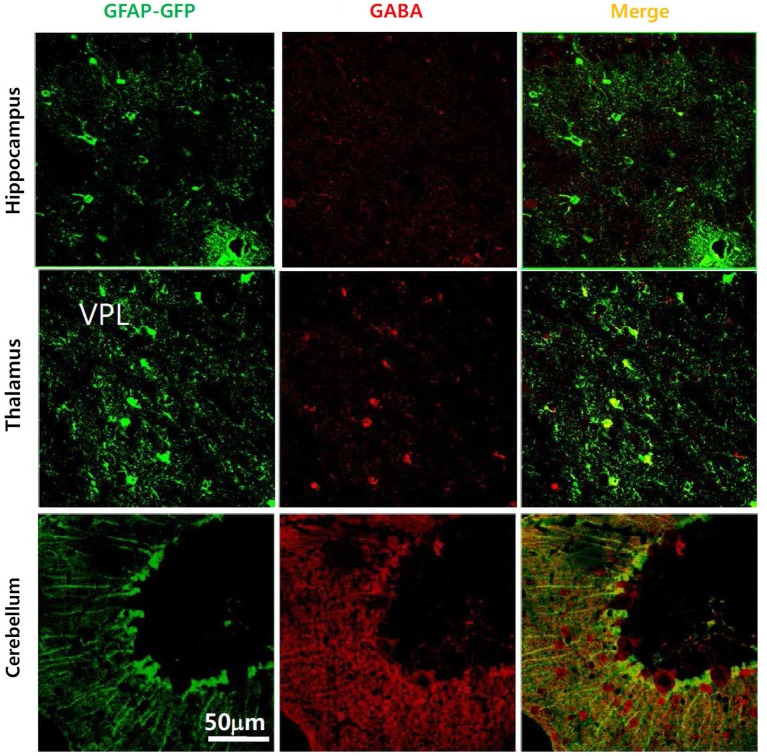
**Presence of GABA in glial cells in various brain regions of GFAP-GFP mice**. Gamma-amino butyric acid is present in the cerebellum, hippocampus and thalamus, though the GABA-containing portion of the glial cell differs between brain regions. Gamma-amino butyric acid does not co-localize much with GFAP-GFP staining in the hippocampus compared to the cerebellar cortex.

Initial reports showed that glia contain considerable amounts of GABA under normal physiological conditions. Astrocytes in brainstem were labeled with an anti-GABA antibody at the level of the soma, in the processes surrounding neurons and in astrocyte endfeet in contact with blood vessels (Blomqvist and Broman, 1988). The presence of GABA in glial cells has been studied extensively in rat optic nerve. In cell cultures of rat optic nerve, 1 week after plating, O2A-oligodendrocyte progenitor cells, type 2 astrocytes and differentiated oligodendrocytes were shown to express GABA (Barres et al., 1990). Gamma-amino butyric acid was localized using immunofluorescence with an astrocytic marker, glial fibrillary acidic protein (GFAP), in developing rat optic nerve from embryonic day 20 to postnatal day 28 (Lake, 1992). A transient increase and gradual decrease of GABA in glial cells after postnatal day 20 was reported by immunostaining and high pressure liquid chromatography (HPLC; Ochi et al., 1993). This study showed the developmental time course of astrocytic GABA expression in rat optic nerve. Gamma-amino butyric acid immunostaining was carried out on cultured astrocytes, and on whole optic nerve and GABA immunoreactivity was localized by GFAP staining. Gamma-amino butyric acid staining was most intense in early neonatal optic nerve and became attenuated over 3 weeks of postnatal development. Staining was pronounced in the astrocyte cell bodies and processes but not in the nucleus. There was a paucity of GABA immunoreactivity by postnatal day 20, both in cultured cells and in whole optic nerve. A biochemical assay for optic nerve GABA using HPLC indicated a relatively high concentration of GABA in the neonate, which rapidly attenuated over the first three postnatal weeks (Ochi et al., 1993). Gamma-amino butyric acid immunoreactivity was also observed in glial cells from the adult rat cerebellum (Martínez-Rodríguez et al., 1993). Both GABA- and Glutamic acid decarboxylase (GAD)-immunoreactivities were observed within dendrites and glial cells using different antisera obtained from rabbits immunized with GABA, baclofen and GAD. These results suggested a possible extrasynaptic release of GABA. However, these early findings had been almost forgotten by brain research scientists until recently (Angulo et al., 2008). We ourselves have recently reported strong GABA immunoreactivities in Bergmann glial cells and lamellar astrocytes of the mouse cerebellum using a commercially available anti-GABA antibody in GFAP-GFP transgenic mice (Lee et al., 2010). We have suggested that the amount of glial GABA is variable depending on different brain regions (Figure 1) and that it positively correlates with the degree of tonic inhibition current (Yoon et al., 2011). For example, glial GABA as evidenced by GABA immunoreactivity was high in the cerebellum and correlated well with a significant level of tonic inhibition current, whereas GABA immunoreactivity was very low in the hippocampus CA1 region with a low level of tonic inhibition current (Yoon et al., 2011). These results suggested that some regulatory mechanism exists to control the level of intracellular GABA in astrocytes. More recently, GABA immunoparticles were localized by electron microscopy in synaptic terminals with symmetric synapses as well as in astrocytes from CA1 and CA3 areas, the stratum radiatum and in the dentate gyrus (Le Meur et al., 2012). This would seem to indicate that a large proportion of hippocampal astrocytes contain the inhibitory transmitter GABA. In addition, immunoreactivity for GABA, GAD65, GAD67 and Bestrophin-1 (Best1), a GABA-permeable channel, has been observed in the meninges and the choroid plexus, implying a non-neuronal source for GABA in the developing mouse brain (Tochitani and Kondo, 2013).

## Production of GABA in glial cells

With respect to the original source of glial GABA, we wonder whether this GABA is synthesized within cells or taken up from the extracellular space. If a glial cell has its own biosynthetic pathway for GABA, then glial GABA content can be modulated by innate molecular factors and can be independent of neuronal activity. However, if glial GABA comes only from uptake of extracellular GABA via GABA transporters, it should be solely dependent on activity-dependent neuronal GABA release, although this explanation might not explain the varying amount of glial GABA in different brain regions (Yoon et al., 2011). Actually, tonic GABA release from glia in cerebellum is known to be independent of neuronal activity and neuronal GABA release (Rossi et al., 2003). Therefore, it is likely that multiple pathways, rather than one single route, are involved in the biosynthesis and regulation of glial GABA.

### Evidence for GAD as a biosynthetic enzyme for glial GABA

Immunoreactivity for the GABA synthesizing enzyme GAD was prominent in neonates but attenuated with development. This was especially the case for GAD67, which was observed in glial cells of neonatal rats but decreased after postnatal day 21. The other isoform, GAD65, was not present in these cells. GABA and GAD are clearly localized in astrocytes of optic nerve and their expression is transient during postnatal development (Ochi et al., 1993). In mature rat cerebellar glia, both GABA and GAD immunoreactivities were found (Martínez-Rodríguez et al., 1993) and more recently, GAD immunoreactivity was reported in astrocyte end feet of the human cerebellar cortex using immunohistochemistry and electron microscopy. In these studies, GAD immunoreactivity was observed in nerve terminals that are in direct contact with the astrocyte perivascular sheath of capillaries and also within astroglial perivascular end feet and endothelial cells (Benagiano et al., 2000). These findings provide further insight into the GABAergic synapse circuitry of the human cerebellar cortex and the detection of “vascular” GAD immunoreactivities suggests that GABAergic mechanisms may regulate cerebellar microvessel function (Benagiano et al., 2000). Glutamic acid decarboxylase was also observed in cultured human astrocytes (Lee et al., 2011b). This work suggested that the intracellular GABA level is about 2.32 mM in untreated cultures of human astrocytes. The authors exported GABA into the culture medium so that an intracellular-extracellular gradient of 3.64-fold was reached. Treatment with inhibitors of the GABA transporters GAT-1, GAT-2, and GAT-3, significantly reduced GABA export in a calcium-independent manner. These findings confirmed the existence of GAD within glial cells but they also emphasized the contribution from GABA transporters in establishing glial GABA content.

### Evidence for monoamine oxidase B (MAOB) as a key biosynthetic enzyme for glial GABA

**Figure 2:**
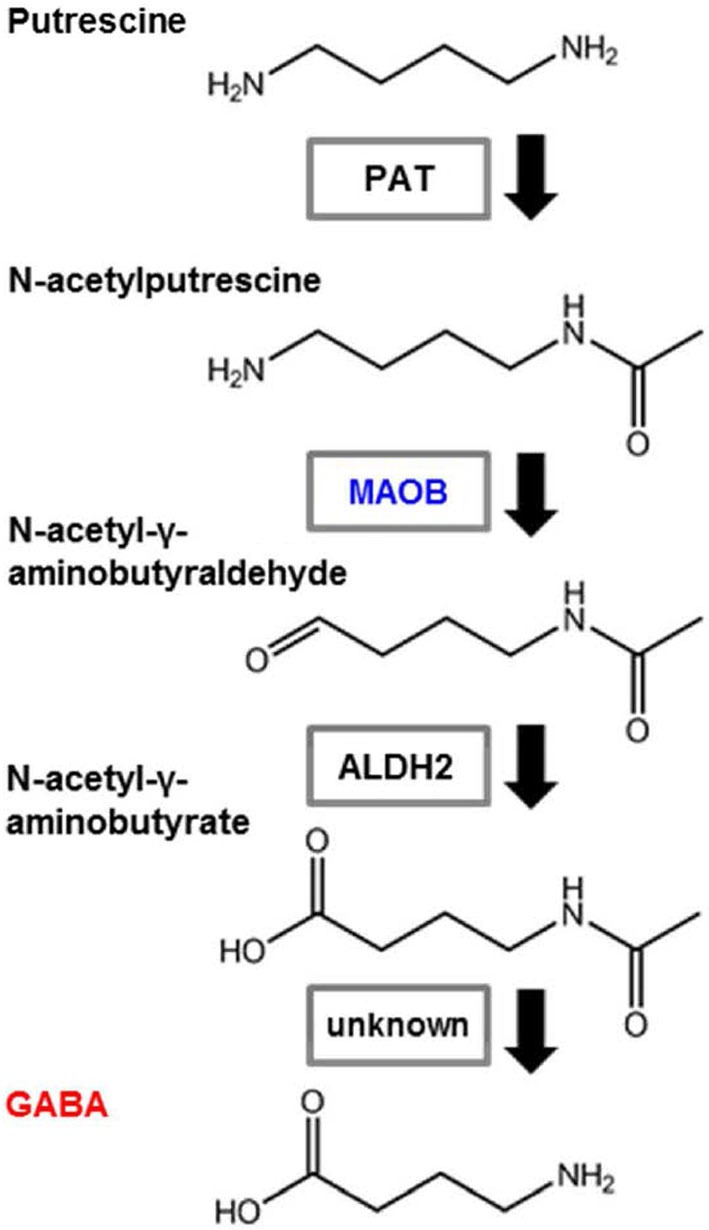
**Pathway showing GABA synthesis from putrescine**. Astrocytes synthesize GABA from putrescine via monoamine oxidation. PAT: putrescine acetyltransferase; MAOB: monoamine oxidase B; ALDH2: aldehyde dehydrogenase 2.

Contrasting reports suggest that GABA is produced by another biosynthetic pathway, which is independent of GAD activity, but that requires putrescine as an initial substrate. This was first suggested in mouse and fish brain (Seiler and Askar, 1971; Seiler et al., 1973). In trout brain, following intraperitoneal and intracerebral injections of [1,4-^14^C]putrescine.2HCl, GABA was shown to be formed *in vivo*, via a pathway that does not have glutamic acid as an intermediate. After intracerebral injections of [1-^14^C] GABA, a half-life of 7 h was obtained for GABA. This slow turnover rate for GABA in trout brain may help to further explain the ineffectiveness of the glutamate decarboxylase inhibitors in lowering the GABA content of fish brain within a few hours (Seiler et al., 1973). Putrescine concentrations can be quantitatively estimated in tissue and have been determined in mouse brain and liver (Seiler and Askar, 1971). This polyamine involved pathway for GABA synthesis was tested in mouse neuroblastoma cells (Kremzner et al., 1975). Polyamine metabolism in cultured mouse neuroblastoma cells was studied with the aim of synthesizing GABA from putrescine and putreanine from spermidine. It was shown that neuroblastoma cells, in the presence of a complete culture medium containing calf serum, readily metabolized [^14^C] putrescine to GABA. The rate of synthesis is similar to that for the synthesis of spermidine from putrescine. In the absence of serum, the conversion of putrescine to GABA is minimal. In the presence of serum, GABA formation is completely inhibited by the diamine oxidase inhibitor aminoguanidine. Synthesized GABA is not readily metabolized to succinate or homocarnosine. Mouse neuroblastoma cells metabolized [^14^C] ornithine to putrescine, GABA, and spermidine. Spermidine was metabolized to putrescine, putreanine and spermine (Kremzner et al., 1975). This is the monoamine oxidase pathway of putrescine (Figure 2). Cultured O2A glial progenitor cells of the optic nerve were able to synthesize GABA from putrescine. In these cells, there is no detectable GAD expression, but a strong GABA immunoreactivity was seen and HPLC measurements revealed an increased quantity of GABA in a putrescine-enriched medium (Barres et al., 1990). Glial cell GABA may also be involved in some pathological conditions. A comparison between GABA formation in primary cultured astrocytes from epileptic and normal mice showed that the rate of GABA production from radioactive putrescine was four times higher in epileptic than in normal mice (Laschet et al., 1992). Moreover, after transiently occluding the carotid arteries of adult gerbils, the GFAP immunoreactivity increased in the damaged forebrain tissue and reactive astrocytes were labeled with GABA, but not with GAD antisera. This GABA immunoreactivity persisted in ischemic animals for up to 3 months without GAD activity (Lin et al., 1993). It has recently been reported that glial monoamine oxidase B (MAOB) is the GABA synthesizing enzyme that mediates tonic GABA release. In the cerebellum and striatum of adult mice, general gene-silencing or knockout of MAOB, or treatment with selegiline, eliminated tonic GABA currents recorded from granule neurons and medium spiny neurons. Glial specific rescue of MAOB resulted in a complete rescue of tonic GABA currents. These results identify MAOB as a key synthesizing enzyme of glial GABA, which is released via the Best1 channel to mediate tonic inhibition in the brain (Yoon et al., 2014).

In summary, there is accumulating evidence for the presence of a GAD or putrescine-derived pathway, suggesting that there are multiple possible routes for the synthesis of glial GABA, as well as the GABA accumulation occurring by uptake through GABA transporters. Until now, most of these findings have been obtained from *in vitro* experiments and cell culture systems. However, to clarify the GABA production mechanism, *in vivo* studies are required to confirm the presence of putrescine and to show that MAOB is a key enzyme for GABA production.

## Release of GABA from glial cells

The synaptic release of GABA and the functional consequences of GABA receptor activation have been extensively studied. The vesicular release of GABA from the axon terminals of inhibitory neurons, which affect nearby postsynaptic targets as well as some extrasynaptic receptors, is a well-established concept of GABAergic signaling. So, questions arising are: Can glial cells release GABA, like neurons? How is glial GABA released? How do glial cells differ in their release of GABA compared to neurons? There is increasing evidence to show that GABA is indeed released from glial cells. Several reports suggest that, unlike neurons, glial GABA can be released via unconventional pathways. For example, GABA may be released from glial cells by the reversal of transporters, or by non-vesicular release mechanisms. We shall review here the recent findings regarding such unconventional mechanisms of glial GABA release and shall discuss the physiological significance of these mechanisms.

### Evidence of GABA release from glial cells

Classically, extracellular GABA was thought to be taken up by GABA transporters expressed on glial cells. Consequently, we would expect glial cells to contain considerable amounts of GABA arising from this uptake. However, several studies provide convincing evidence for the active release of GABA from glial cells. Glioma cell lines have been shown to excrete concentrated GABA into the extracellular medium. This is consistent with the theory that, in the brain, the untransformed counterparts of these glial tumor cells may have the capacity to control the extracellular concentration of neuroactive molecules, especially GABA (Schrier and Thompson, 1974). In cultured hippocampal glial cells, Jow et al. (2004) observed that the conditioned medium contained an inhibitory factor that can hyperpolarize and suppress neuronal activity. They investigated this inhibitory factor using biochemistry, electrophysiology, pharmacology, and mass spectrometry. Mass spectrometry analysis of conditioned medium revealed peaks that are identical to those for GABA. A concentration of up to 500 µM GABA was found in conditioned medium from glial cultures, but no GABA was found in conditioned medium from neuronal cultures. According to these findings, hippocampal glial cells make much more GABA than cortical glia (Jow et al., 2004). To overcome the limitations of culture studies, GABA release from glial cells was then detected and characterized in acute brain slices. In the cerebellum, whole-cell patch clamp recordings of Bergmann glia showed an electrogenic GABA efflux that activated neighboring GABA_A_ receptors (Barakat and Bordey, 2002). These authors suggested that the GABA efflux was mediated by a reversal of the GAT-1 expressed in glial cells (Barakat and Bordey, 2002). In acute slices of olfactory bulb, whole-cell recordings from neurons revealed a spontaneous slow GABA_A_ receptor-mediated current (Kozlov et al., 2006). These studies demonstrated that the release of GABA by astrocytes caused a long lasting and synchronous inhibition of mitral and granule cells in the olfactory bulb (Kozlov et al., 2006). This group also observed that astrocytes are capable of releasing glutamate, as well as GABA, leading to a selective activation of granule cell NMDA receptors. Therefore, they suggested that by releasing both excitatory and inhibitory gliotransmitters, astrocytes are capable of a complex modulatory control of the olfactory network through neuron-glia interactions (Kozlov et al., 2006).

### Mechanism of GABA release from glial cells

Various candidate pathways have been suggested to account for the mechanism of tonic GABA release from glia. Three possibilities seem to be the most likely: (i) vesicular release; (ii) a reversal of GABA transporters; (iii) non-vesicular channel-mediated release. To date however, there is no clear evidence for vesicular release. The lack of GABA-containing synaptic vesicles makes it unlikely that glial cells utilize a vesicular release mechanism. The remaining two possible mechanisms, reversal of transporters and channel-mediated release are the most often suggested. In cerebellar slices, the GABA transporter has been suggested as a candidate mechanism for GABA release (Barakat and Bordey, 2002). It was shown that with 10 mM GABA in the recording pipette, GABA transporters expressed in Bergmann glia operated in the reverse mode, leading to a release of GABA into the extracellular space, subsequently activating GABA_A_ receptors tonically on the same cell. In contrast, the tonic activation of GABA_A_ receptors expressed in cerebellar granule cells of adult rats has been shown to be an action potential-independent and non-vesicular release (Wall and Usowicz, 1997; Mitchell and Silver, 2003; Rossi et al., 2003). Despite this finding, a reversal of the GABA transporter is also suggested by Richerson and Wu (2003) and Lee et al. (2011a). In particular, GAT-2/3 have been considered as an alternative mechanism of GABA release from astrocytes (Héja et al., 2012; Unichenko et al., 2013). Heja et al. demonstrated that GABA is released from astrocytes by the reverse action of glial GABA transporter and that GABA release can be prevented by blocking glutamate uptake with an inhibitor. They argued that the released GABA contributes to the tonic inhibition of neurons in a network activity-dependent manner. Unichenko et al. (2013) reported a role for GAT-2/3 in developmental stages. However, some of these studies were performed with culture systems or under non-physiological conditions. Moreover, the non-vesicular release and action potential-independent release of GABA were shown to contribute to tonic inhibition and tonic GABA_A_ conductance in other brain areas, including the cerebellum, olfactory bulb and hippocampus (Brickley et al., 1996; Kozlov et al., 2006; Glykys and Mody, 2007). Persistent and slow GABA_A_ receptor-mediated excitatory currents activated by a non-synaptic release of GABA have been reported in rat embryonic and early postnatal hippocampal slices. This tonic and slow current was induced by a Ca^2+^- and SNARE protein-independent release of GABA, suggesting a non-conventional and non-vesicular release during development (Demarque et al., 2002). These researchers reported the presence of tonic, spontaneous, and evoked currents in embryonic and neonatal CA1 neurons mediated primarily by the activation of GABA_A_ receptors. These currents persist in the presence of calcium channel blockers or botulinum toxin and are observed in Munc18-1-deficient mice in which vesicular release is abolished. Of interest to note, is that this paracrine communication is modulated by glutamate but not by GABA transporters (Demarque et al., 2002). One study performed in a cell line derived from type 2 astrocytes showed that the release of GABA is sensitive to inhibitors of anion channels (Wang et al., 2002). Recently, we have identified and demonstrated a channel-mediated tonic GABA release from glia (Lee et al., 2010), a non-vesicular and non-conventional mechanism for GABA release in cerebellar slices under physiological conditions. We found that GABA permeates directly through the Ca^2+^-activated anion channel, Best1, to yield GABA release and that tonic inhibition is eliminated by gene silencing of Best1. Using immunohistochemistry, cerebellar Bergmann glial cells and lamellar astrocytes were seen to express both GABA and Best1, and selective expression of Best1 was seen in glial cells. After preventing the general expression of Best1 by Best1 specific shRNA, tonic inhibition was fully rescued by glia specific flanking of the Best1 specific shRNA (Lee et al., 2010).

The precise mechanism underlying tonic GABA release has been difficult to elucidate because this GABA release exhibits several puzzling features that are quite different from those exhibited by the conventional, phasic release of GABA. Our proposed mechanism of channel-mediated release via the Best1 channel can account for each of these properties in cerebellar slices. First, the non-vesicular nature of tonic GABA release is consistent with a channel-mediated mechanism. Second, independence from neuronal activity can be explained by the glial origins of tonic inhibition. Finally, the apparent lack of dependence on external Ca^2+^ arises from substantial activation of Best1 at resting levels of intracellular Ca^2+^, leading to constitutive release of GABA at such intracellular Ca^2+^ levels (Lee et al., 2010). This unprecedented mechanism is consistent with several previous reports that have suggested a non-vesicular and action potential-independent release of GABA (Brickley et al., 1996; Kozlov et al., 2006; Glykys and Mody, 2007) and a well-documented Ca^2+^- and SNARE protein-independent release of GABA during development (Demarque et al., 2002). A further recent report on GABA release from reactive astrocytes in the hippocampus, supports a role for Best1 channels in the release of GABA from glial cells and has implications not only for physiological states but also for pathological conditions (Jo et al., 2014).

A recently published report by Diaz et al. (2011) raised contradictory evidence against our model of Best1-mediated tonic GABA release. However, after examining the paper in detail, we found that the authors provide only pharmacological evidence using NPPB as a blocker of Best1 channel (Diaz et al., 2011). It is important to note that NPPB is notorious for having many side effects. Authors claimed that NPPB enhanced tonic GABA current within 4–5 min, instead of blocking. We also found that NPPB initially increased tonic GABA current, but if we waited longer time period of about 10 min, we saw a block by NPPB (see Figure Sf9a and S9d of Lee et al. (2010)). Thus, if the authors applied NPPB for longer time, they would have seen the block by NPPB, as we observed previously. Nevertheless, this was precisely why we utilized cell-type specific gene-silencing method by lentiviral shRNA (Lee et al., 2010), which is far more specific to Best1 than NPPB. Diaz et al. (2011) did not use any of genetic approaches. Therefore, their claim as shown in the title of their paper, “Best1 channels are insensitive to ethanol and do not mediate tonic GABAergic currents in cerebellar granule cells,” was an overstatement.

## The role of glial GABA release in the brain

### Special roles of glial GABA during development

Depolarization of neurons by astrocytic GABA was first reported in rat embryonic hippocampal neuronal cell cultures (Liu et al., 2000). In this study, neurons exposed to astrocyte-conditioned medium (ACM) showed a highly significant increase in a bicuculline-sensitive persistent GABA_A_ receptor-mediated current, whereas this tonic activation was absent in neurons bathed in normal medium (Liu et al., 2000). These findings implied that astrocyte-released GABA intensifies GABAergic autocrine/paracrine signaling at GABA_A_ receptor/Cl^−^ channels, thus effectively depolarizing differentiating hippocampal neurons near the equilibrium potential of Cl^−^. The same study also found that ACM could rescue neurite outgrowth in embryonic hippocampal and cortical neurons that had been treated with 3-MPA (to block GAD-derived GABA synthesis), via bicuculline- and nitrendipine-sensitive mechanisms. These results indicated that astrocyte-derived GABA provides a critical depolarizing signal that indirectly stimulates Ca^2+^ entry through voltage-gated Ca^2+^ channels, thereby supporting neuritogenesis (Liu et al., 2000). In a separate study, GABA excitation has been shown in rat embryonic and immature hippocampal slices (Demarque et al., 2002). However, during early development, in the marginal zone of mouse neocortex, GABA has been shown to be released via GABA transporters, GAT-2/3, whereas glutamate transporters operate in the uptake mode (Unichenko et al., 2013). This implies that GABA release by glia is essential for development and also that alteration of glial GABA during development can cause neurodevelopmental disorders.

### Inhibitory function of glial GABA in the mature and diseased brain

Previous reports suggest that glial GABA has a special role in the developing brain, though in the more mature brain, glial GABA appears to have an inhibitory effect. Based on numerous studies, we can infer that glial cells must be the source of an inhibitory transmitter responsible for tonic GABA_A_ receptor-mediated currents. For example, glial cells can release GABA into the extracellular space and tonically activate high-affinity GABA_A_ receptors by volume transmission in cerebellum (Rossi et al., 2003; Lee et al., 2010) maintaining a persistent inhibitory tone in these brain regions. Glial GABA has also been seen to act on GABA_B_ receptors (Benedetti et al., 2011). Tonic inhibitory tones have recently been reported in other brain regions including the cortex (Wlodarczyk et al., 2013) and the hippocampus (Pavlov and Walker, 2013; Song et al., 2013). However, phasic GABA released from presynaptic terminals of neurons activates low-affinity synaptic GABA_A_ receptors. The extracellular tonic GABA is estimated to be around 160 nM (Santhakumar et al., 2006; Lee et al., 2010), whereas that of phasic GABA at the synaptic junctions is around 3 mM (Mozrzymas et al., 2003). The synaptically released GABA is constantly taken up by the high performance GABA transporters that are ready for uptake near the synapses. These GABA transporters serve as a barrier that separates the extrasynaptic space and synaptic junctions. The high affinity extrasynaptic GABA_A_ receptors are non-desensitizing and have a half-maximal effective concentration (EC50) of GABA in the range of 0.3–0.7 µM, whereas synaptic GABA_A_ receptors are strongly desensitizing and have an EC50 of GABA in the range of 6–14 µM. There is a clear forty-fold difference in the affinity of GABA_A_ receptors for GABA. To sum up, different locations, extrasynaptic or synaptic, different extracellular concentrations of GABA, and differing degrees of desensitization and sensitivity of GABA_A_ receptors to GABA, make these two distinct GABA_A_ receptor activation modes, tonic and phasic, function quite differently and independently of each other. Therefore, tonic GABA serves to inhibit target neurons on a slow time scale, whereas phasic GABA serves to inhibit target neurons on a fast time scale in the physiological state.

Indeed, tonic inhibition has been reported to have diverse physiological and pathological roles in the CNS. Blocking tonic inhibition with furosemide was shown to increase the firing frequency of cerebellar granule cells (Hamann et al., 2002). Alcohol, by potentiating extrasynaptic GABA_A_, was shown to enhance tonic inhibition in the cerebellum and impair motor behavior (Hanchar et al., 2005). Enhanced tonic inhibition in the thalamus was observed in typical genetic and pharmacological absence seizure models (Cope et al., 2009). Reducing excessive GABA-mediated tonic inhibition was shown to promote functional recovery after stroke in the motor cortex (Clarkson et al., 2010). Alterations in tonic GABA_A_ receptor-mediated signaling was not only found in absence seizure models, but also in temporal lobe epilepsy (Pavlov and Walker, 2013). Very recently, an enduring loss of tonic but not phasic GABA_A_ receptor-mediated currents was shown to critically contribute to the prolonged amygdala disinhibition subsequent to chronic stress exposure (Liu et al., 2014).

There are several lines of evidence to show that GABA release from glia is directly associated with pathological conditions. One recent report found an enhanced tonic inhibition in absence epilepsy and observed a dysfunction in the astrocytic GABA transporter, GAT-1 in absence seizure models (Pirttimaki et al., 2013). Reduced tonic inhibition was reported in mouse models of Huntington’s disease, resulting from a loss of astrocytic GABA release (Wójtowicz et al., 2013). Another study reported anti-inflammatory actions of GABA from glia by modulating microglial activity in human astrocyte cultures (Lee et al., 2011b). More recently, two studies from independent research groups showed that reactive astrocytes abnormally release GABA to impair memory in mouse models of Alzheimer’s disease (Jo et al., 2014; Wu et al., 2014). These recent studies propose a novel and crucial role for glial GABA as the major inhibitory gliotransmitter in many pathological conditions.

## Concluding remarks

This review highlights the compelling evidence accumulating for GABA synthesis and release from glia and reveals that this released GABA plays an important role in brain function. Several mechanisms are proposed for glial GABA biosynthesis and release. Further research is needed to substantiate the precise mechanism for each process. However, it is clear that glial GABA is present in various brain regions and has potentially important roles in each region. Finally, we need to develop new genetic animal models, more detailed characterizations of the various pathological conditions associated with glial GABA and perform more detailed investigations of the regulation of glial GABA in order to elucidate the true nature of the *in vivo* functions of GABA as a gliotransmitter.

## Conflict of interest statement

The authors declare that the research was conducted in the absence of any commercial or financial relationships that could be construed as a potential conflict of interest.
